# Development and validation of TaqMan probe based real time PCR assays for the specific detection of genotype A and B small ruminant lentivirus strains

**DOI:** 10.1186/1746-6148-9-172

**Published:** 2013-09-03

**Authors:** Urška Kuhar, Darja Barlič-Maganja, Jože Grom

**Affiliations:** 1 Veterinary Faculty, Institute for Microbiology and Parasitology, Virology Unit, University of Ljubljana, Gerbičeva 60, SI-1115 Ljubljana, Slovenia; 2 College of Health Care, University of Primorska, Polje 42, SI-6310 Izola, Slovenia

**Keywords:** CAEV, *Gag* matrix, MVV, Real time PCR, Small ruminant lentiviruses

## Abstract

**Background:**

Small ruminant lentiviruses (SRLV) are members of the *Retroviridae* family and infect goats and sheep worldwide. Detection of specific antibodies using AGID and ELISA is the most commonly used means of diagnosing SRLV infection. The most frequent molecular method for detecting the provirus genome is PCR, using peripheral blood leucocytes as target cells. Real time PCR has also recently been used. The aim of this study was to develop a real time PCR for detection of SRLV in order to improve molecular diagnostics of SRLV infections in sheep and goats.

**Results:**

Two new real time PCR assays using TaqMan probes for the specific detection of genotype A (MVV assay) and genoptype B (CAEV assay) SRLV strains and differentiation between them were developed and validated at both analytical and diagnostic levels following MIQE guidelines. The validation results showed that the new real time PCR is 100% specific, with a reliable limit of detection of 26 (CAEV assay) and 72 (MVV assay) plasmid DNA copies, while compared to ELISA the diagnostic sensitivity of both assays was 79% when tested with Slovenian SRLV field samples. Intra-assay and inter-assay coefficients of variation showed overall good repeatability and reproducibility of the new real time PCR assays, except for the highest dilutions.

**Conclusions:**

Two new TaqMan probe based real time PCR assays for the specific detection of genotype A and B SRLV strains and differentiation between them were developed and validated. They can serve as an additional tool for confirming infection with SRLV and may also be useful for early detection of infected animals prior to seroconversion.

## Background

Small ruminant lentiviruses (SRLVs) are members of the *Retroviridae* family and include Caprine arthritis encephalitis virus (CAEV) and Maedi-visna virus (MVV), which infect goats and sheep worldwide [1]. SRLVs cause chronic inflammatory lesions in various organ systems, with the main target organs being the central nervous system, lungs, joints and mammary gland. The clinical disease usually takes years to develop and infection is for life. The infection is mainly transmitted from ewe to lamb through the colostrum and by a respiratory route among animals in close contact [2]. The main target cells are monocytes-macrophages and dendritic cells, in which, following infection, SRLVs integrate as a provirus in the cell genome of the host [3].

The provirus genome of SRLVs is typical of lentiviruses, composed of three genes coding for structural proteins: *gag*, *pol* and *env*, an additional three genes, which encode for non-structural proteins: *tat*, *rev* and *vif* and a non-coding long terminal repeat region (LTR) composed of U3-R-U5 [4].

There is currently no treatment against SRLVs and no successful vaccination is available [1]. Disease control relies on high quality diagnostic tools to identify and eliminate infected animals and to prevent new infections. The agar gel immunodiffusion test (AGID) and, more recently, the enzyme linked immunosorbent assay (ELISA) are the most commonly used means for detecting specific antibodies of a broad spectrum of viral strains and are used as screening assays [1,5]. To confirm or reject the results of the screening assays and to resolve indeterminate results, supplementary tests, such as western blotting (WB) and peptide ELISA are used [6]. Polymerase chain reaction (PCR) is also used to complement the serological methods [1,5]. Due to the slow sero-conversion of infected animals, or even no conversion at all, with the possibility of some animals with a low antibody titre becoming transiently seronegative [7], a combination of serology and PCR might be optimal for detecting SRLV infected animals [5]. Since no free virus has been detected with RT-PCR in the plasma or serum of naturally infected animals, PCR is used mainly for detecting the integrated proviral genome in peripheral blood leucocytes. There have also been a few reports describing real time PCR for the detection of SRLV [8]. The highly heterogeneous SRLV genome and low proviral load hinder the usefulness of PCR for diagnosing infection with SRLV, although the development of an assay based on viral strains circulating in a particular geographic area might solve the former problem [5].

Shah *et al.* (2004) proposed a phylogenetic reclassification that divides SRLV into four genotypes, A to D [9]. A new genotype E has recently been detected [10,11]. Genotypes A and B, MVV and CAEV prototypes, respectively, are widely distributed throughout the world, whereas genotypes C, D and E are geographically restricted. Genotype A is highly heterogeneous, since it contains at least fifteen subtypes, A1 to A15 [9,10,12-15], while genotype B is less complex and contains three subtypes, B1 to B3 [9,16]. Viral strains isolated from Norwegian goats are classified into genotype C and genotype D refers to viral strains isolated in Switzerland and Spain [9,17,18]. Genotype E has only been detected in Italy and it contains two subtypes, E1 and E2 [10,11]. Phylogenetic analysis of SRLVs also supports evidence of natural cross-species transmission [3,15,19], which needs to be considered in disease control.

In order to improve molecular diagnostics of SRLV infections, a two new real time PCR assays were developed, using TaqMan probes for the specific detection of genotype A and B SRLV strains and differentiation between them. Such a method has not to date been described.

## Results

### Real time PCR design

Two new TaqMan probe based real time PCR assays for the specific detection of genotype A and B SRLV strains and differentiation between them were developed: a CAEV assay for specific detection of genotype B SRLV strains and an MVV assay for the specific detection of genotype A SRLV strains. Primers and probes (Table 1) were designed in the *gag*MA gene region to amplify 113 bp (CAEV assay) and 101 bp (MVV assay) long fragments. Both TaqMan probes were labeled with the fluorescent reporter dye FAM and differentiation between MVV and CAEV was only possible using two separate reactions. The same annealing temperatures for both assays enabled simultaneous detection of genotype A and B SRLV strains in the same run but using two separate reactions.

**Table 1:** Primers and probes designed in this study

**Primers and probes**	**Sequence (5′-3′)**	**Positions in genome**	**Amplicon length (bp)**
	MVV assay		
MVVMA F1	GGATACCCCGAGCTCAAAG	520-538*^1^	101
MVVMA R3	TTYAAKGCCCAYAGACARTT	601-620*^1^	
MVV MA	5' FAM-TCTGTCAAGGTCTCCTTCCCG-3' TAMRA	576-596*^1^	
	CAEV assay		
CAEVMA F	GGGAAAAGGGATTATCCTGAG	554-574*^2^	113
CAEVMA R	GTTTTAAGGCACCAYAAACAATTTC	642-666*^2^	
CAEV MA	5' FAM-TCTGTCAAGTKCTCCCCTCTG-3′TAMRA	619-639*^2^	

*^1^ Numbering according to nucleotide sequence of reference SRLV strain MVV KV1514 [GenBank accession number: M10608].

*^2^ Numbering according to nucleotide sequence of reference SRLV strain CAEV Co [GenBank accession number: M33677].

### Analytical and diagnostic specificity

All tested SRLV strains (Table 2) were successfully amplified. The reference virus strain CAEV Co, virus subtype B2 strain and two Slovenian B1 strains were successfully amplified using the CAEV assay. The MVV assay detected the reference virus strain MVV KV1514, virus subtype A3, A4 strains and Slovenian strains of four different subtypes belonging to genotype A (A5, A14, A15 and a genotype A strain 37-88g, which could not be classified into any of the subtypes). When the PCR products were electrophoresed on a 2% agarose gel, they were of specific size (113 bp (CAEV assay) and 101 bp (MVV assay)). The nucleotide sequences of the PCR products amplified with the MVV assay and the CAEV assay were specific for genotype A and genotype B strains, respectively. When the genotype A strains (Table 2) were tested with the CAEV assay and the genotype B strains (Table 2) with the MVV assay for cross reactivity, no reactivity was observed with either of them. No amplification was also observed when samples from seronegative animals from seronegative flocks (Table 3) were tested with both assays. BLAST search results showed significant sequence identity of the primer MVVMA R3 only towards genotype A, while the MVVMA F1 primer showed identity also to genotypes C and E and the MVVMA probe also to genotype E. As shown in the additional file (Additional file 1: Figure S1C), several mismatches of both MVV primers and probe were observed for genotype C. Probe MVVMA showed 100% identity with genotype E and there was one mismatch between MVVMA F1 and genotype E. Five mismatches that were found between the MVVMA R3 primer and genotype E make this primer unable to prime with genotype E. It was estimated that the MVV assay cross reacts with neither genotype C nor genotype E SRLV strains.

**Table 2:** List of virus strains used for optimization and evaluation of analytical specificity of the TaqMan probe based real time PCR assays

**Virus strain**	**Genotype or subtype**	**Country of origin**	**GenBank accession number**
KV1514	A1	Iceland	M10608
20 BAL	A3	Switzerland	/
27 BAL	A3	Switzerland	/
120 M	A3	Switzerland	/
6 BAL	A4	Switzerland	/
94 BC	A4	Switzerland	/
96 BAL	A4	Switzerland	/
CAEV Co	B1	USA	M33677
AghOv478	B2	France	/
1-24g	B1*^1^	Slovenia	HQ910472
1-43g	B1*^1^	Slovenia	HQ910475
1-65g	B1*^1^	Slovenia	HQ910476
1-66g	B1*^1^	Slovenia	HQ910478
1-77g	B1*^1^	Slovenia	HQ910477
2-8g	A14*^1^	Slovenia	HQ910466
2-15g	A14*^1^	Slovenia	HQ910467
2-26g	A14*^1^	Slovenia	HQ910468
2-33g	A14*^1^	Slovenia	HQ910469
2-55g	A14*^1^	Slovenia	HQ910470
31-4s	A15*^1^	Slovenia	JQ611027*^2^
31-9s	A15*^1^	Slovenia	JQ611028*^2^
31-12s	A15*^1^	Slovenia	JQ611029*^2^
31-18s	A15*^1^	Slovenia	JQ611030*^2^
35-1s	A5*^1^	Slovenia	JQ610907
35-44s	A5*^1^	Slovenia	JQ610917
35-49s	A5*^1^	Slovenia	JQ610919
36-10s	A5*^1^	Slovenia	JQ610931
36-14s	A5*^1^	Slovenia	JQ610932
37-20g	B1*^1^	Slovenia	JQ610942
37-25g	B1*^1^	Slovenia	JQ610943
37-31g	B1*^1^	Slovenia	JQ610944
37-63g	B1*^1^	Slovenia	JQ610949
37-65g	B1*^1^	Slovenia	JQ610950
37-88g	A*^1^	Slovenia	JQ610988*^2^

*^1^ according to Kuhar *et al.*[15].

*^2^ sequenced only in *pol* genomic region (Kuhar *et al.*, [15]).

**Table 3:** List of animals used in this study for evaluation of the diagnostic performance of the TaqMan probe based real time PCR assays

**Farm identification**	**Animal species**	**No of animals**	**No (%) of seropositive animals**	**Collection date**	Phylogenetic classification of SRLV strains*^1^
Farm 1	goats	112	99 (88.4%)	January 2008	Genotype B/B1
Farm 2	goats	104	40 (38.5%)	October 2008	Genotype A/A14 and B/B1
Farm 28	sheep	18	/*^4^	March 2011	/
Farm 29	goats	20	/*^4^	March 2011	/
Farm 30	sheep	5	/*^4^	March 2011	/
Farm 31	sheep/goats*^2^	36/3	4 (10.3%)	March 2011	Genotype A/A15
Farm 35	sheep	109	58 (53.2%)	April 2011	Genotype A/A5
Farm 36	sheep/goats*^3^	70/0	39 (55.7%)	April 2011	Genotype A/A5
Farm 37	goats	90	83 (92.2%)	April 2011	Genotype B/B1 and A

*^1^ according to Kuhar *et al.*[15].

*^2^ goats were seronegative.

*^3^ goats were not sampled.

*^4^ all animals were seronegative.

The method is 100% specific, since all tested strains were detected and no amplification was observed when samples from seronegative animals from seronegative flocks were tested. The method enables the detection of genotype A and B SRLV strains, as well as differentiation between them.

### Analytical and diagnostic sensitivity

The analytical sensitivity of the CAEV and MVV assays was evaluated using plasmid DNA carrying CAEV Co DNA and MVV KV1514 DNA, respectively. When 10-fold serial dilutions with plasmid DNA ranging from 1-10^7^ copies/PCR were tested, both assays were able to detect at least 10^2^ copies. The CAEV assay also detected 10 copies with an average Ct value of 39.32, whereas no amplification of 10 copies was observed with the MVV assay (Additional file 2: Table S1). The limit of detection (LOD) of both assays was estimated to be less than 100 copies and was determined after testing ten replicates of plasmid DNA dilutions with 100, 75, 50, 25, 5 and 1 copies/PCR using Probit analysis, which revealed that the CAEV assay reliably detected 26 copies and the MVV assay 72 copies (Additional file 2: Table S2). The diagnostic sensitivity of both assays was tested using samples from ELISA positive animals from 6 seropositive flocks. Samples from 323 ELISA positive animals were tested. The MVV assay detected 81 out of 101 (80.2%) seropositive sheep from farm 31 (infected with A15 SRLV strains) and from farms 35 and 36 (both infected with A5 SRLV strains). The CAEV assay detected 89 out of 99 (89.9%) seropositive goats from farm 1, infected with B1 SRLV strains. Both assays detected 27 out of 40 (67.5%) seropositive goats from farm 2 (infected predominantly with A14 but also with B1 SRLV strains) and 57 out of 83 (68.7%) from farm 37 (infected predominantly with B1 but also with genotype A SRLV strains). Altogether, the two assays detected 254 samples from ELISA positive animals, exhibiting a sensitivity of 79%. The MVV assay also detected all of the 5 samples from ELISA negative animals (1 from farm 2 and 4 from farms 35 and 36) that were LTR-PCR positive in a previous study. These PCR products were electrophoresed on a 2% agarose gel and were of specific size (101 bp). The nucleotide sequences of the PCR products were specific for subtypes A14 and A5. No amplification was observed when samples from 51 ELISA negative animals were tested with the two assays. The kappa statistic was 0.72 (SE 0.03 and 95% confidence interval), which indicates good agreement between ELISA and the two real time PCR assays jointly.

### Real time PCR performance

**Figure 1:**
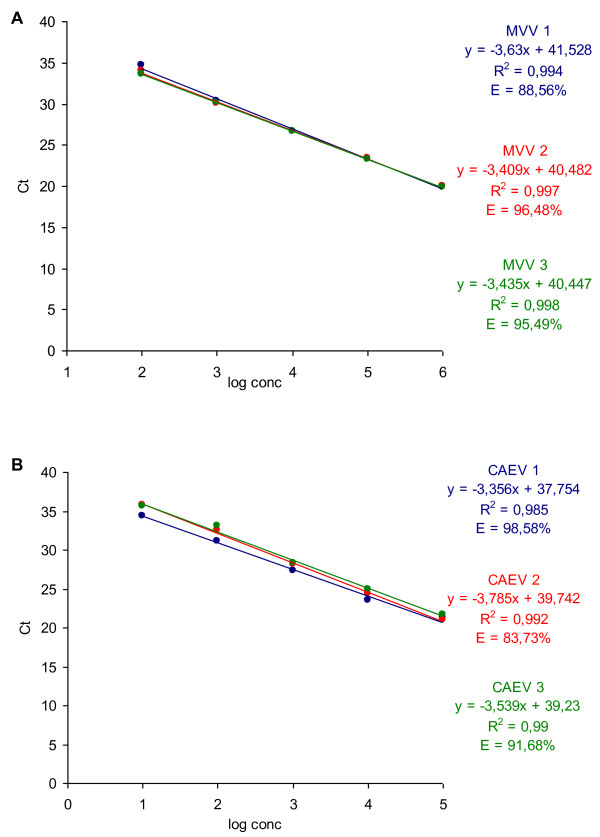
**Standard curves of 3 repeats of the TaqMan probe based real time PCR: (A) MVV assay and (B) CAEV assay.** Ct values of reference virus DNA serial dilutions were plotted against the log value of the target DNA amount (log conc). Regression equations with the coefficient of correlation (R^2^) and efficiency of the reaction (E) are also shown. Each dot represents the result of a triplicate amplification of each dilution.

Serial dilutions of DNA isolated from reference virus strains CAEV Co and MVV KV1514 were prepared to evaluate the performance of the new TaqMan probe based real time PCR assays. Each dilution was amplified in three replicates and repeated in three separate reactions with the CAEV and MVV assays. Intra-assay variability was first tested to determine the repeatability of the two assays. The intra-assay coefficient of variation (CV) of the MVV assay for all serial dilutions ranged from 1.43% to 37.84%, with the most variation observed in the highest dilutions. The CAEV assay exhibited slightly more intra-assay variation than the MVV assay, with the CV for all serial dilutions ranging from 2.3% to 58.26%. Most variation was observed in the undiluted DNA and in the highest dilutions (Table 4). Second, the inter-assay variation was tested to determine the reproducibility of the two assays. The inter-assay CV was from 8.03% to 29.45% and from 13.19% to 36.03% for the MVV and CAEV assays, respectively (Table 5), with the CAEV assay again performing slightly worse. The efficiency, R^2^ and dynamic range were next determined from the standard curves. The reaction efficiencies of all three repeats of the MVV assay were 88.56%, 95.49% and 96.48%, with R^2^ being higher than 0.994 (Figure 1A). All three repeats of the CAEV assay showed reaction efficiencies of 83.73%, 91.68% and 98.58%, with R^2^ being higher than 0.985 (Figure 1B). Both assays had a wide dynamic range of reliable amplification linearity, of at least 5 orders of magnitude (from Ct 19.96 to 34.08 for the MVV assay; from Ct 21.39 to 35.21 for the CAEV assay) (Table 5 and Figure 1).

**Table 4:** Intra-assay performance of the TaqMan probe based real time PCR assays

	**Reaction 1**			**Reaction 2**			**Reaction 3**		
**Theoretical copy number**	**Average Ct**	**Average calculated copy number**	**CV %**	**Average Ct**	**Average calculated copy number**	**CV %**	**Average Ct**	**Average calculated copy number**	**CV %**
MVV assay									
10 ^6^	20	853389	8.67%	20.04	1008108	23.29%	19.83	1007657	7.96%
10 ^5^	23.29	105939	9.25%	23.40	102693	9.62%	23.32	97177	9.14%
10 ^4^	26.69	12190	3.14%	26.66	11365	1.43%	26.64	10483	7.21%
10 ^3^	30.29	1248	6.60%	30.07	1136	5.75%	30.16	990	7.14%
10 ^2^	34.65	83	37.84%	34.01	79	1.35%	33.59	103	34.05%
CAEV assay									
10 ^5^	21.22	87689	38.76	20.96	94514	31.38	21.71	92044	30.09
10 ^4^	23.55	17265	18.81	24.41	11326	17.53	24.91	11230	16.01
10 ^3^	27.33	1463	13.65	28.16	1160	20.64	27.94	1193	3.18
10 ^2^	31.15	66	39.76	32.58	79	17.85	33.06	55	1.84
10	34.4	11	46.69	35.73	13	58.26	35.56	11	2.3

**Table 5:** Inter-assay performance of the TaqMan probe based real time PCR assays

**MVV assay**				**CAEV assay**			
**Theoretical copy number**	**Average Ct**	**Average calculated copy number**	**CV %**	**Theoretical copy number**	**Average Ct**	**Average calculated copy number**	**CV %**
10 ^6^	19.96	956385	15.76%	10 ^5^	21.39	91881	27.61
10 ^5^	23.34	101936	8.93%	10 ^4^	24.38	12775	26.24
10 ^4^	26.66	11223	8.03%	10 ^3^	27.94	1213	13.19
10 ^3^	30.17	1125	11.44%	10 ^2^	32.07	80	35.18
10 ^2^	34.08	88	29.45%	10	35.21	11	36.03

### Control of inhibition

For the control of the potential inhibitory effect of the sample matrix, ten pools of leukocyte suspensions from seronegative animals were spiked with goat synovial membrane (GSM)/lamb synovial membrane (LSM) cells infected with CAEV/MVV. The CV for the CAEV and MVV assays was calculated from the obtained Ct values. Since the CV was 0.2% and 0.42% for the CAEV and MVV assays, respectively, it can be concluded that practically no inhibitors are present in this type of sample matrix.

## Discussion

The use of real time PCR technology in routine microbial diagnostics has rapidly increased, due to its advantages of quick turnaround times, capacity for high throughput and high specificity [20]. Several real time PCR assays for SRLV detection have also been published [8,21-23], but to our knowledge only four real time PCR assays for diagnostic purposes have been described to date [24-27].

Development of a single assay that universally detects SRLV is complicated by the high level of genetic heterogeneity of SRLV, which are grouped into five genotypes and several subtypes [9-18]. Brinkhoff *et al*. (2008) described two universal real time PCR assays for detection of MVV and CAEV, which amplified LTR and leader-*gag* genomic regions using SYBR green intercalating dye. The assays were first tested with SRLV strains from Dutch field flocks and the leader-*gag* PCR performed better, with sensitivities of 88% (sheep) and 82% (goats). When samples from seropositive animals originating from different geographical regions from Norway, France, Spain and Italy were tested, decreased sensitivity was observed, with 85% of positive sheep and 63% of positive goats with the leader-*gag* PCR. However, the phylogenetic classification of the tested SRLV strains was not reported. Firm conclusions concerning the performance of the two assays with international samples were prevented by the small number of investigated samples.

Due to the high heterogeneity of the rapidly evolving lentiviral genome, de Andres *et al.* (2005) suggest that diagnostic methods need to be adapted to viral strains circulating in a particular geographic area. Herrmann-Hoesing *et al*. (2007) developed and validated an OPPV quantitative PCR assay using TaqMan probes, which was designed to amplify a fragment of the TM region of the *env* gene of North American OPPV strains and was tested on naturally infected sheep. Brajon *et al.* (2012) designed a real time PCR assay in the *env* genomic region specific for CAEV, using SYBR green intercalating dye. This assay was tested on naturally infected ELISA positive goats and also on experimentally infected goats. A TaqMan real time PCR assay specific for CAEV was developed in the *gag*CA gene region by Li *et al*. (2013), detecting all AGID positive sheep and goats.

The aim of this study was to develop a TaqMan probe based real time PCR for the specific detection of genotype A (MVV) and B (CAEV) SRLV strains in order to improve molecular diagnostics of SRLV infections. Since the phylogenetic analysis of Slovenian SRLV strains revealed that ovine strains belong to genotype A and caprine strains to genotypes A and B [15,19], two assays were designed for the specific detection of genotype A (MVV assay) and genotype B (CAEV assay) SRLV strains, based mainly on nucleotide sequences from Slovenian strains, as well as nucleotide sequences retrieved from GenBank. Since it is known that SRLV can cross the inter-species barrier [3], it was also designed to differentiate between MVV and CAEV. Such a method has not to date been described. The primers and probes were designed in the *gag*MA gene region. In order to enable detection of a broad range of SRLV strains, degenerate bases were included in both reverse primers and the CAEVMA probe. Since both TaqMan probes were labeled with the fluorescent reporter dye FAM, differentiation between genotype A and B SRLV strains was only possible using two separate reactions. The method was optimized for the simultaneous detection (in the same run but using two separate reactions) of genotype A and B SRLV strains, using the same annealing temperatures for both assays.

It is essential that the assay is performed with high specificity. The analytical and diagnostic specificity of both assays was thus evaluated. Both assays were able to detect all tested phylogenetically diverse SRLV strains belonging to genotype A (A1, A3, A4, A5, A14, A15, strain 37-88g) and genotype B (B1, B2), whereas no amplification was observed when samples from animals from seronegative flocks were tested. When the genotype A strains were tested with the CAEV assay and the genotype B strains were tested with the MVV assay also no amplification was detected. It was established that the method is 100% specific and enables the detection of at least Slovenian genotype A and B SRLV strains, as well as differentiation between them. Although the assays were designed mainly on nucleotide sequences from Slovenian SRLV strains, they were also able to detect A1, A3, A4, B1 and B2 SRLV strains from abroad (Table 2). To validate these PCR assays for international use, extensive studies using international samples should be performed.

Since not only high specificity but also sensitivity of the assay needs to be assured, the analytical and diagnostic sensitivity of both assays was also evaluated. Both assays were highly sensitive, with a reliable limit of detection lower than 10^2^ plasmid copies/PCR, being more sensitive than the assays designed by Brajon *et al*. (2012) and Li *et al*. (2013), who reported 10^2^*env* gene fragments/PCR and 10^2^ copies/μl plasmid DNA, respectively, and slightly less sensitive than the OPPV qPCR assay designed by Herrmann-Hoesing *et al*. (2007), which detected from 1 to 6 × 10^4^ copies/μg DNA in naturally infected sheep. On the other hand, the new real time PCR assays performed with less sensitivity when tested with Slovenian SRLV field samples, since they jointly detected only 79% of samples of ELISA positive animals. Although the sensitivity of each assay separately could not be determined, since two flocks (farms 2 and 37) were infected with both genotypes A and B and not all SRLV strains from seropositive animals were genotyped, based on the results from single strain infected flocks, the CAEV assay was more sensitive. A decreased sensitivity of both assays was observed in flocks with dual infections. A firm conclusion concerning the sensitivity of each separate assay was thus not possible. Similar sensitivities were obtained by Brinkhoff *et al*. (2008) and Brajon *et al*. (2012), whereas Herrmann-Hoesing *et al*. (2007) reported a 96.2% positive concordance of the OPPV qPCR. However, comparing the new real time PCR assays with conventional LTR-PCR, which detected SRLV in 54% of ELISA positive animals (unpublished results, Kuhar U.), they showed significantly better diagnostic performance. The poor diagnostic sensitivity of PCR assays for SRLV detection is due to the low virus load *in vivo* and the SRLV strain variation [5]. Although in this study an attempt was made to overcome the latter with assays designed on the basis of SRLV nucleotide sequences from the Slovenian strains under investigation, a firm conclusion on whether the resulting diagnostic sensitivity was low due to the strain variation or the low virus load, was not possible since the sensitivity of the assays using plasmids corresponding to different field strains was not examined. Nevertheless, the MVV assay also detected all 5 samples from ELISA negative animals that were LTR-PCR positive in a previous study (unpublished results, Kuhar U.) Good agreement between ELISA and both real time PCR assays jointly was obtained with the kappa statistic 0.73, which is similar to the agreement for sheep samples with the leader-*gag* PCR reported by Brinkhoff *et al*. (2008), whereas Herrmann-Hoesing *et al*. (2007) reported excellent agreement (kappa value 0.93).

The use of real time PCR in a diagnostic laboratory also requires the assay to be repeatable and reproducible. The new real time PCR assays showed a good level of repeatability and reproducibility, except for the highest dilutions, with an estimated input of virus DNA copies equal or below 10^2^. The CAEV assay exhibited slightly worse repeatability and reproducibility than the MVV assay. Intra-assay and inter-assay coefficients of variation of both assays were comparable to the OPPV qPCR assay reported by Herrmann-Hoesing *et al*. (2007).

Following the validation guidelines proposed by Bustin *et al.* (2009) [28], the efficiency of the reaction, together with R^2^ and the dynamic range, was also determined to evaluate the performance of the assays. The efficiency slightly varied for the two assays and, in one repeat of the two assays, showed values below optimal in relation to quantitative assays [29]. Although the efficiency of both assays was not always as optimal as recommended for quantitative methods, it is still satisfactory if the method is used simply for the detection of SRLV positive animals. Since both assays were developed to detect a broad range of SRLV strains, which was only possible using degenerate bases in primers and one probe, optimal performance, as recommended for quantitative methods aiming at a specific target, cannot be expected. The R^2^ values of both assays were higher than 0.985, which indicates a good correlation between the amount of template and the Ct values [30]. Both assays also had a wide dynamic range of at least 5 orders of magnitude. Since the two viruses could not be cultured in a higher titre, it could not be determined whether the dynamic range is wider.

In disease control, the price of a diagnostic procedure is crucial. PCR and real time PCR methods for SRLV detection in general are hampered by the expensive and time consuming DNA extraction procedure from peripheral blood leucocytes. A further disadvantage of the new developed real time PCR procedure is the need to use two reactions for genotyping and the detection of both genotypes A and B. However, the cost was reduced by using 15 μl of reaction mix. Nevertheless, both assays can be performed in the same run due to the same amplification conditions, thus reducing the time required. Furthermore, this report follows the (MIQE) guidelines proposed by Bustin *et al.* (2009), which ensure the integrity and experimental transparency of studies that are based on quantitative real time PCR protocols. None of the previously published reports describing diagnostic real time PCR assays for detection of SRLV have met these criteria. The performance of both assays designed by Brinkhoff *et al*. (2008) was evaluated only in terms of efficiency, while the limit of detection, intra-assay and inter-assay variance were not reported. Experiments in relation to efficiency, intra-assay and inter-assay variance were not carried out by Brajon *et al*. (2012) and Li *et al*. (2013). The efficiency of the OPPV qPCR by Herrmann-Hoesing *et al*. (2007) was also not reported.

## Conclusions

Two new TaqMan probe based real time PCR assays for the specific detection of genotype A and B SRLV strains and differentiation between them were developed and validated. Validation experiments followed the (MIQE) guidelines to ensure the integrity and experimental transparency of development of the new assays. The validation results showed that the assays are not only highly specific and sensitive but also repeatable and reproducible. The two new TaqMan probe based real time PCR assays can serve as an additional tool for confirming infection with SRLV and may also be useful for early detection of infected animals prior to seroconversion.

## Methods

### Animals and blood samples

A total of 567 animals were examined in this study to evaluate the diagnostic performance of the new TaqMan probe based real time PCR and are listed in Table 3. Samples were taken on a voluntary basis. Blood samples from all animals were taken by jugular venipuncture for both serum and whole blood collection, using 10 ml vacutainers without anticoagulant and 10 ml EDTA vacutainers, respectively. Samples were stored at −20°C until further use, unless peripheral blood leucocytes (PBL) were isolated from whole blood as previously described [19]. Sera samples from the animals investigated were tested for the presence of specific antibodies using a commercial ELISA assay Chekit-CAEV/MVV Screening ELISA Test Kit (IDEXX Laboratories) according to the manufacturer’s instructions. Genomic DNA was extracted from peripheral blood leucocytes as previously described [19].

### Virus strains

Virus strains used for optimization and evaluation of the analytical specificity of the new TaqMan probe based real time PCR are listed in Table 2. Reference virus strains CAEV Co and MVV KV1514, subtype A3, A4 virus strains, which were kindly provided by Dr. Giuseppe Bertoni (Institute of Veterinary Virology, University of Bern, Switzerland), subtype B2 virus strain, which was kindly provided by Dr. Stephen Valas (Anses, Niort Laboratory, Niort, France) and phylogenetically diverse Slovenian SRLV strains [15,19] were used in this study. Reference virus strains CAEV Co and MVV KV1514 were cultivated on goat synovial membrane (GSM) and lamb synovial membrane (LSM) cell cultures, respectively, and used for evaluating the performance of the new TaqMan probe based real time PCR assays.

### Nucleotide sequencing

Altogether, 51 sequences of the *gag*MA gene region from Slovenian SRLV strains were determined in this study using previously described PCR procedures and nucleotide sequencing [19]. Nucleotide sequencing was also used for confirmation of the specificity of PCR products and performed as previously described [19]. All novel sequences of the *gag*MA gene region from Slovenian SRLV strains reported in this study were deposited in GenBank [GenBank accession numbers: JQ610905-JQ610955].

### Primer and probe design

The primers and TaqMan probes were designed on the basis of aligned nucleotide sequences in the *gag*MA gene region. Two assays were designed: a CAEV assay and an MVV assay. The nucleotide sequences from 36 Slovenian CAEV strains (B1) [GenBank accession numbers: HQ910472-HQ910493 and JQ610942-JQ610955] together with 11 CAEV strains retrieved from GenBank [GenBank accession numbers: DQ190014 (B1), DQ190016 (B1), DQ190019 (B1), DQ190020 (B1), DQ190028 (B1), DQ190033 (B1), DQ190044 (B1), M33677 (B1), AY900630 (B1), FJ195346 (B2) and AY265456 (B2)] were aligned with the Clustal X program [31]. Multiple alignments were also created from nucleotide sequences of 49 Slovenian MVV strains (A5 and A14) [GenBank accession numbers: HQ910460-HQ910471 and JQ610905-JQ610941] and 9 MVV strains retrieved from GenBank [GenBank accession numbers: AY445885 (A4), DQ084347 (A3), M10608 (A1), AY101611 (A2), M31646 (A1), EU010123 (A1), AF479638 (P1OLV), EU010125 (A8) and AY265455 (A9)]. The most conserved regions for primers and probes were manually identified and Oligo Analyzer software (Integrated DNA Technologies, USA) that is available online [32] was used to evaluate the kinetics of primers and probes. Particular attention was devoted to ensuring the same kinetics of amplification of both amplicons, to allow simultaneous amplification under the same PCR conditions. Multiple alignments with corresponding primers and probes are presented in an additional file (Additional file 1: Figure S1A and S1B).

The specificity of primers and probes was tested *in silico* with a Basic Local Alignment Search Tool (BLAST) of public databases. The forward primer CAEVMA F, the reverse primer CAEVMA R and the probe CAEVMA were designed for the specific detection of genotype B SRLV strains with the CAEV assay. Three forward primers, 3 reverse primers and probe MVVMA were initially designed (not shown) for the specific detection of genotype A SRLV strains with the MVV assay and, after optimization described below, the forward and reverse primers with the optimal performance, namely forward primer MVVMA F1 and reverse primer MVVMA R3, were selected. According to BLAST search results, the primers and probe of the CAEV assay showed significant sequence identity only towards genotype B, whereas primer MVVMA F1 also showed identity to genotypes C and E and probe MVVMA also showed identity to genotype E. Therefore an alignment of genotype C [GenBank accession number: AF322109], E [GenBank accession number: EU293538] and the primers and probe of the MVV assay was created (Additional file 1: Figure S1C).

TaqMan probes were labeled with the fluorescent reporter dye FAM (6-Carboxyfluorescein) at the 5’end and with the fluorescent quencher dye TAMRA (6-Carboxytetramethylrhodamine) at the 3’end. Primers and probes were produced at Sigma-Aldrich (St. Luis, USA).

The primer and probe sequences are listed in Table 1.

### Optimization of real time PCR

The new TaqMan probe based real time PCR assays were performed on an ABI PRISM 7000 SDS (Applied Biosystems, USA). Amplification was carried out using isolated DNA with Platinum® Quantitative PCR SuperMix-UDG with ROX (Invitrogen, USA) in a total volume of 15 μl.

For the optimization of the MVV assay, the reference virus strain MVV KV1514 and Slovenian SRLV strains of four different subtypes belonging to genotype A were used. For the optimization of the CAEV assay, the reference virus strain CAEV Co and two B1 strains from Slovenia were used (Table 2). The annealing temperature (52°C, 54°C, 56°C in 58°C) was first optimized. For each assay, the matrix titration method was applied to optimize the concentration of primers and probe using a fixed amount of template and reaction system. Various primer (200-800 nM) and probe (50-200 nM) concentrations and combinations were tested. The conditions for optimal amplification were determined based on the lowest cycle threshold (Ct) value and the highest increase in fluorescence (ΔRn). For the MVV assay, all combinations of primers that were initially designed were tested and the combination that amplified all tested virus strains, and considering the aforementioned criteria, was selected (Table 1).

### Real time PCR setup

The reaction mix for the CAEV assay was composed of 7.5 μl 2 × Platinum® Quantitative PCR SuperMix-UDG with ROX buffer, 600 nM of forward primer, 400 nM of reverse primer, 200 nM of probe, 2 μl of sample DNA and DEPC H_2_O to reach the final 15 μl reaction volume. For the MVV assay, 800 nM of each primer and 150 nM of probe were used.

Thermocycling conditions were 2 min at 50°C, followed by initial denaturation at 95°C for 2 min and 45 cycles at 95°C for 15 s (denaturation) and 60 s at 56°C (annealing and extension).

A non-template control (DEPC H_2_O; NTC) and a positive control (isolated DNA of reference virus strains CAEV Co and MVV KV1514; PC) were included in each run. Each sample of DNA, NTC and PC was tested in duplicate.

The results were analyzed using SDS 1.2 software (Applied Biosystems, USA).

### Validation procedures

In order to ensure confidence that the real time PCR assay performs consistently and reliably when implemented in a diagnostic setting, validation at both analytical and diagnostic levels was necessary. The new TaqMan probe based real time PCR assays were validated according to “minimum information for publication of quantitative real time PCR experiments” (MIQE) guidelines for the publication of quantitative real time PCR experiments suggested by Bustin *et al.* (2009), also considering the OIE recommendations for validation of PCR methods used for the diagnosis of infectious disease [28,33].

### Analytical specificity

The analytical specificity was evaluated with the reference virus strain CAEV Co (subtype B1), the reference virus strain MVV KV1514 (subtype A1), virus subtype A3, A4, B2 strains and Slovenian strains of four different subtypes belonging to genotype A (A5, A14, A15 and a genotype A strain 37-88g, which could not be classified into any of the subtypes), together with two Slovenian B1 strains (Table 2). The PCR products were electrophoresed on a 2% agarose gel, stained with ethidium bromide and visualized under UV light. The corresponding 113 bp (CAEV assay) and 101 bp (MVV assay) fragments were cut out, purified and sequenced in both directions for confirmation.

For cross reactivity, the genotype A strains (listed in Table 2) were tested with the CAEV assay and the genotype B strains (listed in Table 2) were tested with the MVV assay in one repeat, using two wells for each virus strain.

### Analytical sensitivity

#### Construction of DNA standards

Two plasmid standards were prepared using PCR products. The PCRs were performed as previously described [19], using isolated DNA of reference virus strains CAEV Co and MVV KV1514 with primers GIN5/GIN3 and MVV1/MVV3, respectively. The PCR products were electrophoresed in a 1.8% agarose gel, stained with ethidium bromide and visualized under UV light. The corresponding 512-bp and 482-bp fragments were cut out, purified and sequenced in both directions for confirmation. Purified CAEV Co and MVV 1514 DNAs were cloned into competent DH5α (*Escherichia coli*) cells via the pCR 2.1 vector, which was supplied by a TA Cloning® Kit (Invitrogen, USA). All procedures followed the manufacturer’s instructions. Plasmids with the correct inserts were purified using QIAprep Spin Miniprep (Qiagen, Germany) according to the manufacturer’s instructions and sequenced using the aforemen-tioned primers to confirm the correct sequence. The concentration of DNA was measured with a Qubit® Fluorometer (Invitrogen, USA). The copy number of the plasmid DNA with the insert DNA was calculated with a formula that is available online [34]:

$$\mathrm{Number}\;\mathrm{of}\;\mathrm{copies}=\frac{6.022\times 10^{23}\;\left(\mathrm{copies}/\mathrm{mol}\right)\times \mathrm{DNA}\;\mathrm{amount}\;\left(\mathrm{ng}\right)}{\mathrm{DNA}\;\mathrm{length}\;\left(\mathrm{bp}\right)\times 10^{9}\;\left(\mathrm{ng}/g\right)\times 650\;\left(g/\mathrm{mol}/\mathrm{bp}\right)\;}$$

### Detection limit (LOD)

First, 10-fold serial dilutions of the plasmid DNA carrying the CAEV Co DNA and of the plasmid DNA carrying the MVV KV1514 DNA were tested. Each 10-fold dilution of 1-10^7^ copies/PCR was amplified in three replicates. A larger number of replicates around the endpoint were next tested for determining the LOD. The plasmid DNAs were diluted into 100, 75, 50, 25, 5 and 1 copies/PCR and ten replicates of each dilution were tested. Probit analysis was performed using the SPSS software package version 20, to calculate the LOD with 95% probability.

### Efficiency of the reaction, repeatability and reproducibility

Ten-fold serial dilutions using isolated DNA of reference virus strains CAEV Co and MVV KV1514 cultivated on GSM and LSM cell cultures, respectively, were tested to evaluate the performance of the new TaqMan probe based real time PCR assays. Four consecutive 10-fold dilutions, starting with undiluted DNA, were amplified in three replicates and repeated in three separate reactions. A standard curve was constructed for each reaction on the basis of DNA serial dilutions, on which Ct values were plotted against the log value of the target DNA amount (log conc). The initial copy number in undiluted DNA was estimated by comparing the Ct values of plasmid standard dilution with known copy number with the undiluted DNA.

The efficiency (E) of the reaction was calculated using the formula: E = (10^(1/s)^) – 1, where *s* is the slope of the linear regression line. The correlation coefficient (R^2^) was also evaluated.

The coefficient of variation (CV) for copy number variance, using the calculated copy number, was used to evaluate the repeatability (intra-assay variance) and reproducibility (inter-assay variance) of the reaction. The calculated copy number was calculated from the Ct values of DNA dilutions using regression equations obtained from the standard curve.

### Control of inhibition

Reference virus strains CAEV Co and MVV KV1514 were cultivated on GSM and LSM cell cultures, respectively. Ten pools of goat and sheep leucocytes suspension from animals originating from seronegative and LTR-PCR negative flocks were prepared. The infected GSM and LSM cells were spiked separately into the pool suspensions at a ratio of 1:10. Five pools were spiked with the infected GSM cells and five pools with infected LSM cells. DNA was extracted from 200 μl of each pool as previously described [13] and was used for the new TaqMan probe based real time PCR assays. The Ct values were analyzed and the CVs were calculated from Ct values.

### Diagnostic specificity

Forty-three whole blood samples from animals originating from 3 seronegative and LTR-PCR negative flocks (flock 28, flock 29 and flock 30) (Table 3) were tested with the new TaqMan probe based real time PCR assays. All samples were tested with the CAEV assay and MVV assay.

### Diagnostic sensitivity

A total of 379 animals from 6 seropositive flocks (Table 3) were tested with the new TaqMan probe based real time PCR assays, namely 323 samples from ELISA positive animals and 5 samples from ELISA negative but LTR-PCR positive animals, with the latter samples having been tested in a previous study (unpublished results, Kuhar U.). Altogether, there were 524 animals in seropositive flocks. All ELISA positive animals were investigated and, in addition, 51 out of 201 samples from ELISA negative animals were also tested in order to establish whether the new TaqMan probe based real time PCR assays detect SRLV DNA in additional ELISA negative animals. The sample size of seronegative animals was calculated according to the method described by Thrusfield (1995), so that the detection of at least one seropositive animal was possible with a probability of 95% and considering the expected seroprevalence [35]. Instead of the expected seroprevalence, the expected percentage of seronegative and PCR positive animals was used and was assumed to be 5% [8]. Sheep samples were tested with the MVV assay and goat samples were tested with the CAEV assay. If the phylogenetic analysis of SRLV strains revealed the presence of genotype A and B virus strains in one flock, samples from seropositive goats that were negative with the CAEV assay were also tested with the MVV assay and seropositive sheep that were negative with the MVV assay were also tested with the CAEV assay. Kappa statistics were obtained to evaluate agreement between ELISA and the PCR assays.

## Competing interests

The authors declare that they have no competing interests.

## Authors’ contributions

UK designed the study, carried out the laboratory work, compiled and analysed the data, and drafted the manuscript. JG and DBM helped to design the study and supervised the study. All of the authors have critically revised and approved the final manuscript.

## Supplementary Material

Additional file 1: Figure S1 Multiple sequence alignments of: (A) genotype A and (B) genotype B SRLV strains at primer and probe binding sites. (C) Sequence alignment of genotype C and E SRLV strains with MVV assay primers and probe. Alignments start at nucleotide positions 505 (A) of MVV KV1514 (M10608) and 548 (B) of CAEV Co (M33677). Sequences of genotype C and E SRLV strains are aligned with MVV assay primers and probe, with denoted mismatches between virus sequences and primers (probe) (C). Reverse complement of virus sequences aligned with MVVMA R3 primer and MVVMA probe are shown. Click here for file

Additional file 2: Table S1 Assay performance of the TaqMan probe based real time PCR on plasmid DNA. **Table S2**. Positive replicates of plasmid DNA copies around the endpoint of the TaqMan probe based real time PCR for calculating LOD by Probit analysis. Click here for file
