# Supplementary material for: Development and validation of TaqMan probe based real time PCR assays for the specific detection of genotype A and B small ruminant lentivirus strains
Source: BMC Vet Res. 2013 Sep 3;9:172. doi: 10.1186/1746-6148-9-172 (PMC3766269; doi:10.1186/1746-6148-9-172)
Supplement: Additional file 1: Figure S1 — Multiple sequence alignments of: (A) genotype A and (B) genotype B SRLV strains at primer and probe binding sites. (C) Sequence alignment of genotype C and E SRLV strains with MVV assay primers and probe. Alignments start at nucleotide positions 505 (A) of MVV KV1514 (M10608) and 548 (B) of CAEV Co (M33677). Sequences of genotype C and E SRLV strains are aligned with MVV assay primers and probe, with denoted mismatches between virus sequences and primers (probe) (C). Reverse complement of virus sequences aligned with MVVMA R3 primer and MVVMA probe are shown. [file 1746-6148-9-172-S1.pdf]

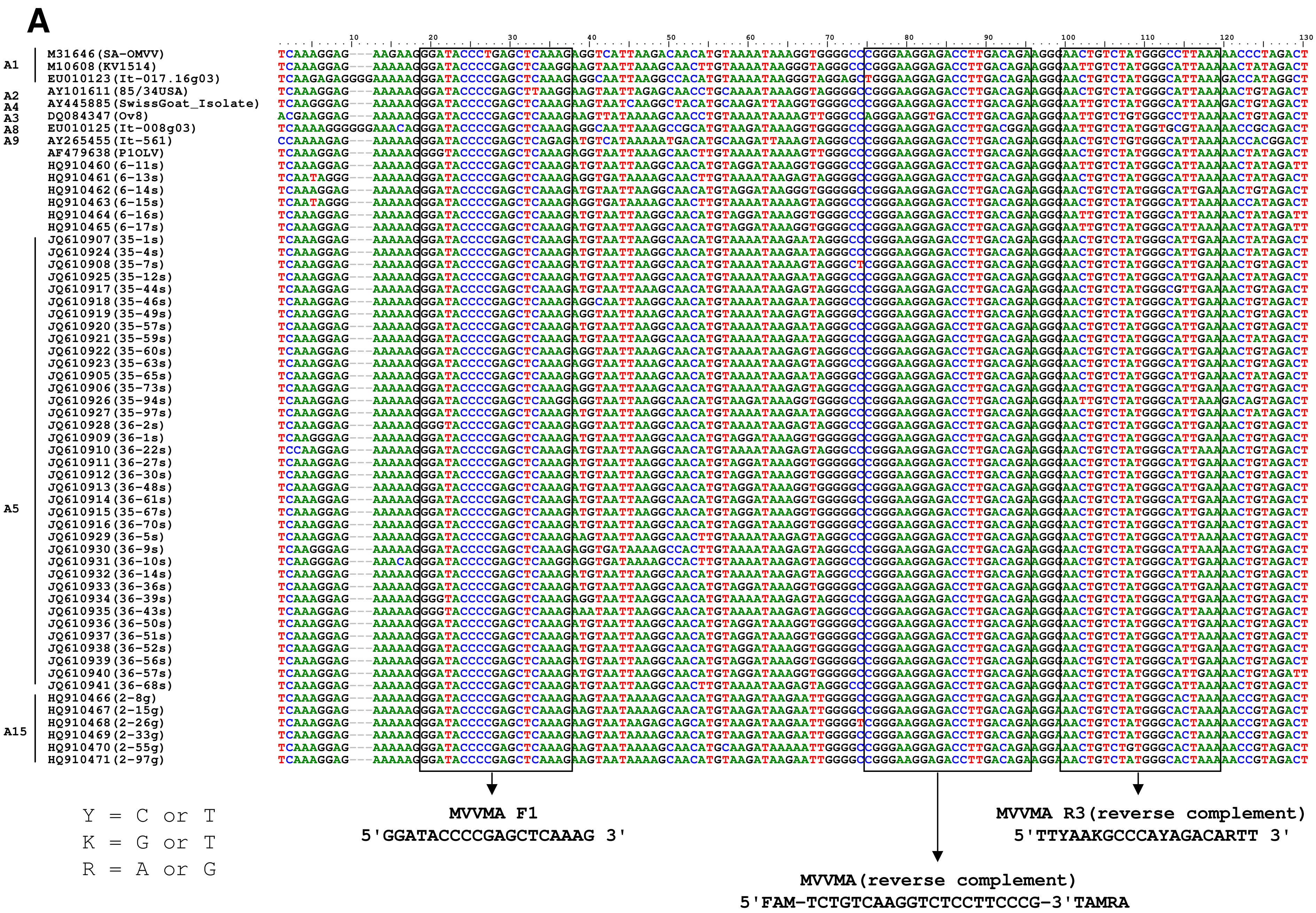
A
A1
A2
A4
A3
A8
A9
A5
A15
MVVMA F1
5'GGATACCCCGAGCTCAAAG 3'
MVVMA R3(reverse complement)
5'TTYAAKGCCCAYAGACARTT 3'
MVVMA(reverse complement)
5'FAM-TCTGTCAAGGTCTCCTTCCCG-3'TAMRA
Y = C or T
K = G or T
R = A or G

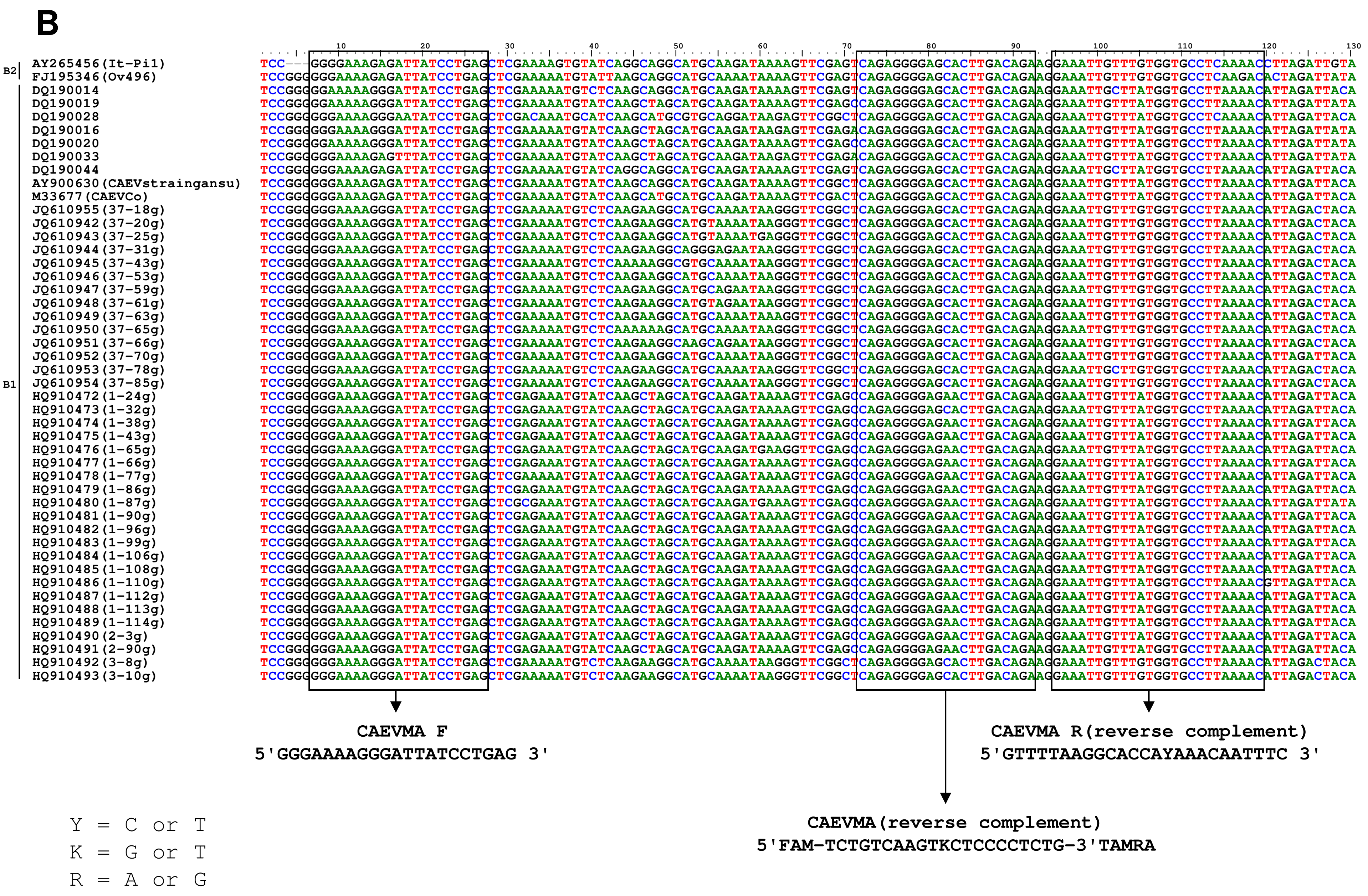
B
B2
AY265456(It-Pi1)
FJ195346(Ov496)
B1
DQ190014
DQ190019
DQ190028
DQ190016
DQ190020
DQ190033
DQ190044
AY900630(CAEVstraingansu)
M33677(CAEVCo)
JQ610955(37-18g)
JQ610942(37-20g)
JQ610943(37-25g)
JQ610944(37-31g)
JQ610945(37-43g)
JQ610946(37-53g)
JQ610947(37-59g)
JQ610948(37-61g)
JQ610949(37-63g)
JQ610950(37-65g)
JQ610951(37-66g)
JQ610952(37-70g)
JQ610953(37-78g)
JQ610954(37-85g)
HQ910472(1-24g)
HQ910473(1-32g)
HQ910474(1-38g)
HQ910475(1-43g)
HQ910476(1-65g)
HQ910477(1-66g)
HQ910478(1-77g)
HQ910479(1-86g)
HQ910480(1-87g)
HQ910481(1-90g)
HQ910482(1-96g)
HQ910483(1-99g)
HQ910484(1-106g)
HQ910485(1-108g)
HQ910486(1-110g)
HQ910487(1-112g)
HQ910488(1-113g)
HQ910489(1-114g)
HQ910490(2-3g)
HQ910491(2-90g)
HQ910492(3-8g)
HQ910493(3-10g)
CAEVMA F
5'GGGAAAAGGGATTATCCTGAG 3'
CAEVMA R(reverse complement)
5'GTTTTAAGGCACCAYAAACAATTTC 3'
CAEVMA(reverse complement)
5'FAM-TCTGTCAAGTKCTCCCCTCTG-3'TAMRA
Y = C or T
K = G or T
R = A or G

**C**

```
                                            10        20        30
                                   ....|....|....|....|....|....|....|...
AF322109(CAEV1GA)genotypeC         TCCGGAAGGGGAAAGGAGTACCCCGAGCTAAAAGAATG
primer MVVMA F1                    ---------------GGATACCCCGAGCTCAAAG----

EU293538(Roccaverano)genotypeE     TCAGATGGGAGAAAGGGATACCCCGAACTCAAAGAATT
primer MVVMA F1                    ---------------GGATACCCCGAGCTCAAAG----
```

```
                                            10        20        30        40        50
                                   ....|....|....|....|....|....|....|....|....|....|..
AF322109(CAEV1GA)genotypeC         TTTTTATACACCAGAGACAATTTCCTTCTGTCAGGCGCTCCCCCCCAGCCCT
probe MVVMA                        --------------------------TCTGTCAAGGTCTCCTTCCCG-----
primer MVVMA R3                    --TTYAAKGCCCAYAGACARTT------------------------------

EU293538(Roccaverano)genotypeE     TTTTTATACACCAAAGACAATTGCCTTCTGTCAAGGTCTCCTTCCCGACTCC
probe MVVMA                        --------------------------TCTGTCAAGGTCTCCTTCCCG-----
primer MVVMA R3                    --TTYAAKGCCCAYAGACARTT------------------------------
```

Y = C or T
K = G or T
R = A or G
