# Supplementary material for: Development and validation of TaqMan probe based real time PCR assays for the specific detection of genotype A and B small ruminant lentivirus strains
Source: BMC Vet Res. 2013 Sep 3;9:172. doi: 10.1186/1746-6148-9-172 (PMC3766269; doi:10.1186/1746-6148-9-172)
Supplement: Additional file 2: Table S1 — Assay performance of the TaqMan probe based real time PCR on plasmid DNA. Table S2. Positive replicates of plasmid DNA copies around the endpoint of the TaqMan probe based real time PCR for calculating LOD by Probit analysis. [file 1746-6148-9-172-S2.doc]

## Table S1. Assay performance of the TaqMan probe based real time PCR on plasmid DNA.

| Copy number/PCR reaction | Average Ct MVV assay | Average Ct CAEV assay |
| --- | --- | --- |
| 10 ^7^ | 14.65 | 15.12 |
| 10 ^6^ | 18.68 | 18.05 |
| 10 ^5^ | 24.45 | 22.06 |
| 10 ^4^ | 28.09 | 27.19 |
| 10 ^3^ | 32.55 | 31.05 |
| 10 ^2^ | 34.51 | 34.71 |
| 10 | NA* | 39.32 |

*NA: no amplification

## Table S2. Positive replicates of plasmid DNA copies around the endpoint of the TaqMan probe based real time PCR for calculating LOD by Probit analysis.

| Copy number of plasmid DNA | Number of replicates | Number of positive replicates (% of positives) | |
| --- | --- | --- | --- |
|  |  | CAEV assay* | MVV assay* |
| 100 | 10 | 10 (100%) | 10 (100%) |
| 75 | 10 | 10 (100%) | 9 (90%) |
| 50 | 10 | 10 (100%) | 7 (70%) |
| 25 | 10 | 9(90%) | 6 (60%) |
| 10 | 10 | 4 (40%) | 0 (0%) |
| 5 | 10 | 1 (10%) | 0 (0%) |
| 1 | 10 | 0 (0%) | 0 (0%) |

*The LOD determined by Probit analysis with 95% probability was 26 copies of plasmid DNA for CAEV assay (95% confidence interval, 20 to 42 copies) and 72 copies of plasmid DNA for MVV assay (95% confidence interval, 52 to 155 copies).
